# Expression of proteins in *Escherichia coli* as fusions with maltose-binding protein to rescue non-expressed targets in a high-throughput protein-expression and purification pipeline

**DOI:** 10.1107/S1744309111022159

**Published:** 2011-08-13

**Authors:** Stephen N. Hewitt, Ryan Choi, Angela Kelley, Gregory J. Crowther, Alberto J. Napuli, Wesley C. Van Voorhis

**Affiliations:** a Seattle Structural Genomics Center for Infectious Disease (SSGCID), University of Washington, WA 98195, USA; b Division of Allergy and Infectious Diseases, School of Medicine, Box 356423, University of Washington, Seattle, WA 98195-6423, USA

**Keywords:** structural genomics, high throughput, maltose-binding protein, MBP fusion, protein expression, SSGCID

## Abstract

Despite recent advances, the expression of heterologous proteins in *Escherichia coli* for crystallization remains a nontrivial challenge. The present study investigates the efficacy of maltose-binding protein (MBP) fusion as a general strategy for rescuing the expression of target proteins. From a group of sequence-verified clones with undetectable levels of protein expression in an *E. coli* T7 expression system, 95 clones representing 16 phylogenetically diverse organisms were selected for recloning into a chimeric expression vector with an N-terminal histidine-tagged MBP. PCR-amplified inserts were annealed into an identical ligation-independent cloning region in an MBP-fusion vector and were analyzed for expression and solubility by high-throughput nickel-affinity binding. This approach yielded detectable expression of 72% of the clones; soluble expression was visible in 62%. However, the solubility of most proteins was marginal to poor upon cleavage of the MBP tag. This study offers large-scale evidence that MBP can improve the soluble expression of previously non-expressing proteins from a variety of eukaryotic and prokaryotic organisms. While the behavior of the cleaved proteins was disappointing, further refinements in MBP tagging may permit the more widespread use of MBP-fusion proteins in crystallographic studies.

## Abstract 2

The rescue of protein-expression levels by cloning genes into MBP-fusion vector is described.

## 1. Introduction

The process of solving three-dimensional protein structures by X-ray crystallography is a multi-stage effort with unique challenges at each step. The initial challenge is expression of the protein in a sufficient quantity and with sufficient purity to obtain diffraction-quality crystals. Typically, recombinant proteins are expressed in *Escherichia coli* with small affinity tags such as hexahistidine (His tag) to allow efficient separation of the desired protein by affinity chromatography (Arnold, 1991 ▶). *E. coli* expression systems are a proven and cost-effective method of pro­ducing large quantities of high-quality recombinant proteins (Arnold, 1991 ▶; Gathmann *et al.*, 2006 ▶; Mus-Veteau, 2002 ▶). However, this approach is often insufficient for soluble expression of recombinant protein. The Structural Genomics Center has estimated that up to 50% of all prokaryotic proteins are insoluble when expressed in *E. coli* (Edwards *et al.*, 2000 ▶). Failure to express soluble eukaryotic proteins is even more common; for instance, reports indicate that only 6–20% of human and *Plasmodium* proteins are solubly expressed using standard expression methods (Stevens, 2000 ▶; Mehlin *et al.*, 2006 ▶). Insoluble protein expression is thus a major impediment to structural genomics efforts.

One strategy for increasing expression and solubility is fusing the target protein to a large affinity tag such as glutathione *S*-transferase (GST), thioredoxin (TRX) or maltose-binding protein (MBP) (Smith, 2000 ▶; Sachdev & Chirgwin, 2000 ▶; LaVallie *et al.*, 2000 ▶; Kapust & Waugh, 1999 ▶). Of these, the most promising may be MBP. Although unaltered MBP can be purified using a cross-linked amylose resin affinity matrix, addition of a His tag or GST improves the yield and purity (Pryor & Leiting, 1997 ▶). His-tagged MBP-fusion con­structs therefore allow metal-affinity purification strategies combined with the potential for increased solubility (di Guan *et al.*, 1988 ▶).

Previous studies have provided preliminary evidence of the ability of MBP to rescue the expression and solubility of proteins. In a com­parison of three macromolecule chimeric constructs, MBP was far more effective than GST or TRX in solubilizing six notoriously insoluble proteins (Kapust & Waugh, 1999 ▶). In a study of 32 small human proteins expressed in *E. coli* in tandem with MBP, researchers observed increased solubility and expression in 19 out of 32 con­structs compared with His-tag expression (Hammarström *et al.*, 2002 ▶). In an examination of membrane proteins from *Mycobacterium tuberculosis*, the expression of 16 out of 22 proteins was rescued by fusion to MBP and 13 of these 16 were soluble (Korepanova *et al.*, 2007 ▶). A larger study by Kataeva *et al.* (2005 ▶) observed increased soluble expression levels in 60 out of 66 *Clostridium thermocellum* and 38 out of 79 *Shewanella oneidensis* proteins when targets were expressed fused to MBP in combination with decreased induction temperatures, compared with the expression of GST or NusA fusion partners. To our knowledge, however, a large-scale study of the effects of MBP on proteins from diverse eukaryotic and prokaryotic organisms has yet to be reported.

The goal of this study was to examine fusion proteins on a scale permitting a highly accurate assessment of the rescue rate of MBP. We attempted MBP-mediated rescue of 95 His-tagged targets from diverse sources. These targets had no soluble or total expression but were sequence-validated, ensuring that the lack of expression was not attributable to an incorrect target or an empty vector.

## 2. Methods

**Figure 1:**
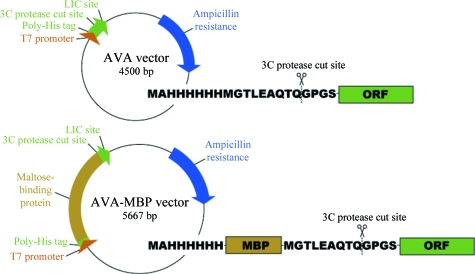
Comparison of AVA vector and AVA-MBP vector.

Targets for the SSGCID pipeline were selected for their predicted essentiality, virulence factor and general potential as drug targets. For cloning, targets were amplified from either purified genomic DNA or cDNA using standardized primers containing sequences specific for the cloning site followed by a sequence complementary (adjusted to 331 K *T*_m_) to the template gene: FWD primer 5′-GGGTCCTG­GTTCATG…-3′ and REV primer 5′-CTTGTTCGTGCTGTTTA­TTA…-3′ (Invitrogen). PCR conditions were optimized depending on the G+C content of the template sequence. A typical reaction mixture was as follows: 35.2 µl dH_2_O (Sigma, catalog No. W3513), 5 µl Expand High Fidelity Buffer (10×) with 15 m*M* MgCl_2_ (Roche), 0.4 µl 25 m*M* dNTPs (Qiagen, catalog No. 201912), 0.4 µl Expand High Fidelity Enzyme Mix (Roche, catalog No. 11732641001), 4 µl (10 µ*M*) FWD primer, 4 µl (10 µ*M*) REV primer and 1 µl (10 ng) of the respective template DNA. PCR amplicons were run on a 1% agarose gel to verify the expected size of the amplified gene; the band was then excised from the gel and extracted from the agarose using a QiaQuick kit (Qiagen, catalog No. 28181). The purified PCR product was cloned into expression vector AVA0421 (which expresses protein with a hexahistidine-tag fusion that is cleavable with 3C protease to leave a minimal four-amino-acid sequence at the N-­terminus) or AVA-MBP (which expresses protein with an N-terminal hexahistidine tag in tandem with MBP that is cleavable with 3C protease) by ligation-independent cloning (LIC; Aslanidis & De Jong, 1990 ▶; Fig. 1 ▶). Briefly, purified PCR product was treated with T4 polymerase in the presence of the single nucleotide dATP, creating overhangs, and then annealed with compatible, linearized and T4-treated AVA0421 vector (Mehlin *et al.*, 2006 ▶). Annealed vector and insert were transformed into NovaBlue competent cells (Novagen, catalog No. 71011-4) and plated on LB agar (BD Difco LB Agar Miller; BD, catalog No. 244520) with 50 µg ml^−1^ each of ampicillin (Anatrace, catalog No. A1000) and carbenicillin (Duchefa Biochemie, catalog No. C0109.0025) to select for cells carrying the expression plasmid. The presence of the insert was verified by colony PCR (using the above conditions but the colony was resuspended in water and used as template instead of purified DNA). Plasmid DNA was purified (QIAprep Turbo mini-prep kit; Qiagen, catalog No. 27191) from 1 ml overnight cultures and then transformed into the expression host Rosetta Oxford [BL21 Star(DE3)-R3-pRARE2] (Choi *et al.*, 2011 ▶).

For protein purification, 2 l cultures of the clone were grown in a LEX bioreactor (Harbinger) at 293 K in auto-induction medium (Studier, 2005 ▶). After 72 h of growth, the culture was pelleted (Sorvall RC 12BP fitted with an H-12000 rotor; spun for 20 min at 4300 rev min^−1^) and the cell paste was harvested and flash-frozen in liquid nitrogen. To prepare protein samples, the cell paste was solubilized in 200 ml lysis buffer (25 m*M* HEPES, 500 m*M* NaCl, 5% glycerol, 30 m*M* imidazole, 0.5% CHAPS, 10 m*M* MgCl_2_, 1 m*M* TCEP, 25 µg ml^−1^ AEBSF pH 7.0) with 0.01 g lysozyme and sonicated for 30 min (100 W, cycles of 15 s pulse-on and 15 s pulse-off; Virtis, catalog No. 408912). After sonication, the samples were treated with benzonase (500 U) and then centrifuged for 1 h (14 000 rev min^−1^ in a Sorvall SLA-1500 rotor) to clarify the cell debris. The protein was purified by immobilized metal ion-affinity chromatography on pre-equilibrated (25 m*M* HEPES pH 7.0, 500 m*M* NaCl, 5% glycerol, 30 m*M* imidazole, 1 m*M* TCEP and 0.025% azide) 5 ml Ni Sepharose columns (HisTrap FF; GE Healthcare, catalog No. 17-5255-01) using an ÄTKAexplorer. After thorough washing, the bound protein was eluted from the nickel column by addition of elution buffer (25 m*M* HEPES pH 7.0, 500 m*M* NaCl, 5% glycerol, 1 m*M* TCEP, 250 m*M* imidazole and 0.025% azide). Fractions from nickel-affinity chromatography were analyzed for protein content and pooled. The N-­terminal 6×His tag was removed by treatment with His-MBP-3C protease overnight at 277 K in 3C buffer (25 m*M* HEPES pH 7.0, 200 m*M* NaCl, 5% glycerol, 1 m*M* TCEP, 0.025% azide). Cleaved protein samples were passed over Ni resin beads (Ni Sepharose 6 Fast Flow; GE Healthcare, catalog No. 17-5318-02) to remove noncleaved protein, the cleaved 6×His tag, 3C protease and contaminants that bind to nickel. Clarified cleaved protein was then further purified by size-exclusion chromatography (SEC; HiLoad 26/60 Superdex 75; GE Healthcare, catalog No. 17-1071-01) using an ÄTKAprime to collect fractions in SEC buffer (25 m*M* HEPES pH 7.0, 500 m*M* NaCl, 5% glycerol, 2 m*M* DTT, 0.025% azide). SEC fractions were analyzed by SDS–PAGE. The highest intensity SEC fractions were pooled and concentrated (Amicon Ultra-15 concentrator with a molecular-weight cutoff of 3000 Da; Fisher, catalog No. UFC901096). Protein samples were aliquoted in 100 µl volumes, flash-frozen in liquid nitrogen and stored at 193 K.

## 3. Results and discussion

As part of the SSGCID structural genomics pipeline, we routinely analyze by SDS–PAGE both the total and soluble fractions of small-scale cultures in 96-well sets (see Choi *et al.*, 2011 ▶) in order to identify tractable targets for purification. These high-throughput screens are analyzed to identify insoluble or non-expressing constructs and, in most cases, to remove them from the pipeline. However, the present study further pursued these cases of suboptimal expression in order to directly compare His-tag-fusion expression with MBP-fusion expression. Our specific goal was to quantity the frequency with which adding MBP to a protein increased expression and solubility sufficiently to allow purification. We modified our His-tag *E. coli* expression vector to include an MBP tag between the hexahistidine residues and the 3C cleavage recognition site (allowing cleavage of the N-terminal tag during purification) four amino acids upstream of the methionine start signal of the target protein, while maintaining the same insertion sequence (Fig. 1 ▶). This design strategy allowed us to efficiently employ the same PCR-amplified T4 polymerase-treated product for LIC insertion into either vector without requiring further modification.

From the entire set of constructs screened for the SSGCID project in our standard expression vector with the minimal His tag, we identified 497 unique clones that had neither total nor soluble protein expression. From this group of nearly 500 targets, we verified by sequencing that 295 of the constructs contained the expected gene sequence, eliminating the possibility of an empty vector as the reason for the lack of protein expression and verifying correct PCR amplification. 95 of these genes were selected for further study based on our desire to cover a wide range of protein functions from both prokary­otic and eukaryotic organisms (see Supplementary Material^1^ for a complete list of proteins).

**Figure 2:**
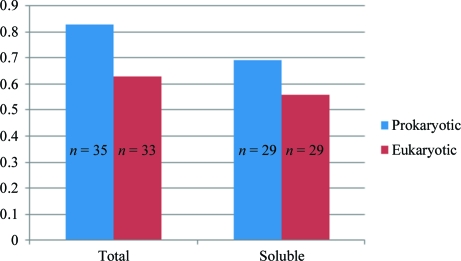
Distribution of protein solubility by species, grouped by prokaryotic and eukaryotic kingdoms.

Of the 95 targets selected for this study, 94 were successfully cloned into the expression host and screened for solubility. The vast majority of the targets (72%) expressed some level of protein of the expected size (the combined size of the target protein and MBP) as visualized by SDS–PAGE. 58 targets (62%) additionally had protein of the expected size in the soluble fraction. The majority (37) of these had detectable but low soluble expression (band easily visible on the gel), 11 samples had medium solubility (representing roughly 20–40% of soluble protein) and ten had high soluble expression levels (representing ≥40% of the soluble protein). Of the 36 remaining targets, 23 expressed a protein band which corresponded to the expected size of MBP alone, indicating that in these samples MBP was solubly expressed but not the fusion partner. Rescue rates were fairly similar among targets from prokaryotic and eukaryotic sources (Fig. 2 ▶).

**Figure 3:**
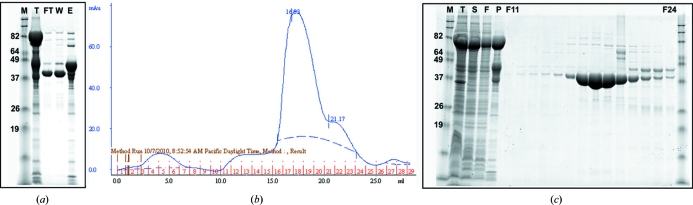
SDS–PAGE and SEC chromatogram of an exemplary rescued target which was easily purified. Even after cleavage and removal of the His-MBP tag, the target protein remained soluble and expressed at the expected size. (*a*) SDS–PAGE of samples from initial IMAC purification and subsequent 3C cleavage of the His-MBP tag. P represents pure sample after the first IMAC step; the observed molecular weight corresponds to the expected size of the recombinant protein expressed with fused MBP. After cleavage with 3C protease, His-MBP is retained on subsequent IMAC (E), while flowthrough (FT) and wash (W) samples contained protein that passes over the nickel column unbound. Unbound recombinant protein was pooled and subjected to SEC. (*b*) Chromatogram of SEC indicating fractions and sieving properties of smaller molecular-weight protein (without MBP tag). (*c*) SDS–PAGE of SEC fractions showing the purity of the final preparation. M, molecular-weight marker; T, sample from total lysate; S, sample from soluble fraction after centrifugation. The protein expressed and purified was an uncharacterized protein from *Coccidioides immitis*.

**Figure 4:**
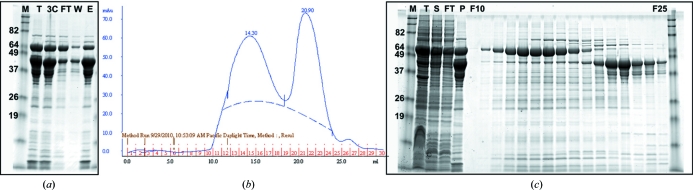
Example of more typical protein-purification products. Large quantities of fused protein running at the molecular weight of target protein and MBP combined are visible in the total (T) and soluble (S) fractions. (*a*) After cleavage, two forms of the protein remain visible: a band corresponding to the size of MBP and a band corresponding to the size of MBP plus the target protein; very little to no target protein remains in solution. A chromatogram (*b*) and SDS–PAGE (*c*) of SEC fractions from uncleaved sample indicate a heterogenous solution of either the recombinant protein expressed with MBP (higher molecular-weight band) or MBP alone (lower molecular-weight band). M, molecular-weight marker; T, total lysate; S, soluble fraction; FT, flowthrough from IMAC after 3C cleavage; W, wash after 3C cleavage; E, eluate with 250 m*M* imidazole from IMAC after 3C cleavage. The protein expressed and purified was *Brucella abortus* blue (type 1) copper protein.

To date, purifications have been attempted on 21 of the 58 solubly expressing proteins. 15 of these purifications yielded >1 mg purified protein; in 12 cases the abundance and purity of the target sample were considered to be adequate for crystallization trials (Fig. 3 ▶) and in one case crystals suitable for X-ray diffraction were obtained. This low success rate (obtaining crystals from only one of 21 attempted purifications) reflects the fact that the target proteins were usually of poor solubility after the MBP tag had been removed. Even in the 12 best purifications mentioned above yields of the MBP-free target protein were low (often 1–10 mg) and they were sometimes con­taminated with significant amounts of still uncleaved protein (Fig. 4 ▶).

Insolubility may be an intrinsic property of a particular protein or may be a consequence of inadequate folding properties of the expression host. Our observations are consistent with MBP being a transiently stabilizing protein, with partner proteins falling out of solution once MBP is removed. One model is that highly soluble MBP acts as a chaperone by sequestering the aggregation-prone passenger protein, allowing native conformational folding of the nascent protein but in a weak reversible manner (Kapust & Waugh, 1999 ▶). Recent studies have made available new fusion constructs with mutations to MBP to decrease the surface entropy and increase the rigidity of the polypeptide sequence linking MBP to the recombinant protein (Moon *et al.*, 2010 ▶). These modifications have allowed direct structure solution by X-ray crystallography and molecular replacement without necessitating the removal of the MBP tag (Smyth *et al.*, 2003 ▶; Moon *et al.*, 2010 ▶). Although further advancements need to be made for this to be viable as a high-throughput rescue strategy, this approach shows promise for those recombinant protein products that could not be separated from the MBP tag by 3C cleavage. Other solubility-enhancing tags, such as SUMO expression systems, are a potential alternative for rescue of insoluble or non-expressing recombinant constructs (Yunus & Lima, 2009 ▶).

## Supplementary Material

Complete breakdown of all the targets analyzed for this study; includes the maximum status for each target at the time of article submission.. DOI: 10.1107/S1744309111022159/en5465sup1.xls

## Footnotes

1 Supplementary material has been deposited in the IUCr electronic archive (Reference: EN5465).
